# Beauty and the busy mind: Occupied working memory resources impair aesthetic experiences in everyday life

**DOI:** 10.1371/journal.pone.0248529

**Published:** 2021-03-12

**Authors:** Rosalie Weigand, Thomas Jacobsen

**Affiliations:** Experimental Psychology Unit, Helmut Schmidt University / University of the Federal Armed Forces Hamburg, Hamburg, Germany; University of Wuerzburg, GERMANY

## Abstract

Aesthetic experiences have been distinguished from other experiences based on an aesthetic mode of processing that often entails concentrating working memory resources on the aesthetic stimulus. Since working memory is a limited-capacity system, there should be a trade-off between available resources and the aesthetic experience. To test whether the intensity of the aesthetic experience is reduced if working memory resources are otherwise occupied, we employed an experience sampling method. One hundred and fifteen undergraduate students (45% female; *M*_*age*_ = 23.50 years, *SD* = 2.72 years) participated in a 2-week experience sampling study and furnished a total of 15,047 reports of their aesthetic experiences. As measures of current working memory resources, participants answered questions regarding their current working memory load and whether they were engaged in a second task. In addition, they reported whether they had had an aesthetic experience and how much they had savored the aesthetic experience. Multilevel modeling was used for data analysis. A higher working memory load was associated with fewer aesthetic experiences and reduced the savoring of aesthetic experiences. Second tasks, however, that were perceived as demanding and requiring a lot of concentration enhanced the savoring of aesthetic experiences. In sum, other goal-oriented behavior that requires working memory resources appears to conflict with aesthetic experiences in everyday life.

## Introduction

The experience of beauty can make us stop and savor the present moment. It can provide small breaks from everyday hustle and bustle and can even cause peak experiences. Aesthetic experiences refer to the evaluative reception of an object or sensorial entity with respect to one or more relevant concepts (e.g., beauty or elegance [1]) and are positively associated with well-being and prosociality [2]. Aesthetic experiences have different qualities: For instance, we can reflect upon the aesthetic value of an entity (i.e., engage in aesthetic contemplation), come to a decision about it (i.e., make an aesthetic judgment), or involuntarily switch our attention to the aesthetic processing of a stimulus (i.e., experience aesthetic distraction [3]).

Since aesthetic experiences arise in response to a variety of domains, it is necessary to consider the mental mode of processing involved in this “exceptional state of mind” [4](p.2) in order to gain a comprehensive understanding of aesthetic experiences [5]. For this purpose, several researchers have distinguished aesthetic experiences from other experiences based on an “aesthetic mode.” The aesthetic mode comprises a disinterested interest, which entails an attentional focus on the stimulus, integrating context, memory, and sensory qualities, and neglecting self-referential concerns or everyday life perceptions [6–8]. As a possible (evolutionary) reason for entering this mode, the “stopping for knowledge” hypothesis proposes that aesthetic concepts (e.g., beauty) can act as signals to the system to stop acting for the purpose of learning something new [9]. In general, the mental mode of processing concerns how information is represented in working memory [10]. Because one of the central limits of human cognition is the restricted amount of information that can be kept in working memory [11], and variations in “working memory capacity” [12] (p.614), that is working memory resources, are associated with variations in several important abilities, including control of attention, the question naturally arises: How is the emergence and intensity of aesthetic experiences affected if working memory resources are depleted?

Aesthetic experiences are coupled with enhanced attentional focus towards sensory stimulation and a concentration of working memory resources on the object of aesthetic appreciation [9, 13]. The ability to focus attention enhances the processing of aesthetic attributes, leading to heightened engagement and pleasure [14, 15]. Since this condition of focused attention fully engages the perceiver’s working memory resources [16], theoretical accounts suggested that the instinct to attend to aesthetic stimuli is activated only when the individual is not otherwise involved in some goal-oriented behavior [9]. This suggests that depleted working memory resources might completely prevent the perceiver from entering an aesthetic mode. In the laboratory, active working memory depletion has been found to reduce the understanding of artworks [17] and the beauty felt from beautiful stimuli [18]. This points in a similar direction, but rather suggests an attenuated version of the relationship between working memory resources and aesthetic experiences. The aesthetic experience might be impaired but not completely prevented.

The main goal of the present work was to assess the effect of depleted working memory resources on aesthetic experiences in people’s natural environments. Besides the obvious difficulty of capturing aesthetic experiences at all in an artificial laboratory environment, laboratory research particularly complicates measurement of the quality of aesthetic distraction. Therefore, we used an experience sampling method (ESM) to assess how depleted working memory resources affect the frequency and intensity of aesthetic experiences. ESM is particularly well suited to complement laboratory studies of the aesthetic experience due to its ecological validity and high temporal and contextual resolution [19]. Whereas previous research has focused mainly on the art domains, we aimed at a domain-general investigation. Also, while laboratory studies have actively manipulated working memory resources by engaging participants in established tasks known to target working memory, we wanted to know how the working memory load (i.e., the used amount of working memory resources) imposed by everyday tasks affected aesthetic experiences. Finally, we wanted to improve predictive validity by measuring the intensity of the aesthetic experience with an established scale instead of relying on one-item measurements. Since aesthetic emotions are key outcome variables of aesthetic experiences [1, 6, 14], we chose to measure an aesthetic emotion. For this purpose, we chose savoring as a “time-tested model of aesthetic emotion” [20](p. 1) that refers to the appreciation and extensive processing of personal emotional information in aesthetic contexts. Savoring involves clear focusing on the experience [21]. It requires detachment from pragmatic concerns and action, on the one hand, and immersion in the current aesthetic experience, on the other [22]. Therefore, savoring can be considered a direct indicator of the intensity of the aesthetic experience.

We predicted that any depletion of working memory resources in everyday life would negatively affect aesthetic experiences. First, we predicted that a working memory load would reduce the probability of having an aesthetic experience (**Hypothesis 1**). Specifically, we tested whether an individual’s perceived overall working memory load is associated with a reduced probability of aesthetic experiences. Second, we predicted that a working memory load would reduce the savoring of aesthetic experiences (**Hypothesis 2**). Specifically, we tested whether the presence of a second task as well as the individual’s perceived overall working memory load is associated with reduced savoring. Third, since everyday tasks vary greatly in the demands they impose on working memory, we wanted to take some specific task characteristics that require more working memory resources [23] into account. We predicted that certain task characteristics impair aesthetic experiences (**Hypothesis 3**). Specifically, we tested whether second tasks that are challenging or interesting, require concentration, or are perceived as important impair savoring of aesthetic experiences.

## Materials and methods

### Sample

Potential participants were approached via a university mailing list. We collected data from 116 undergraduates at the Helmut Schmidt University/University of the Federal Armed Forces Hamburg who participated in our study in exchange for partial fulfillment of course requirements. Since one data set was excluded from further analysis because the file was unreadable, we collected usable data from 115 subjects (52 female, 63 male) aged 20 to 34 years (*M* = 23.50, *SD* = 2.72). On average, subjects completed 135.5 (*SD* = 21.5, range = 39 to 164) usable experience-sampling questionnaires. Across the participants, ratings for 15,047 occasions were recorded.

Our target sample size was based on several considerations.

First, simulation studies have shown that in multilevel linear modeling, 50 or more Level 2 units are necessary to accurately estimate standard errors [24], and the most stable solutions are obtained if the number of Level 2 units lies between 50 and 100 [25] and the sample size at Level 1 is between 30 and 50 responses for each group [24]. Second, in multilevel logistic modeling, a minimum of 50 Level 1 units and 40 Level 2 units are needed to accurately estimate small fixed effects (odds ratio [*OR*] = 1.70) with small intercept variance (*var* ≈ 0.1), whereas 100 Level 1 units and 80 Level 2 units are needed when there is an interest in cross-level interactions and/or a large intercept variance (set at *var* ≈ 0.5 [26]). With 115 Level 2 units and an average of 135.5 Level 1 units, our sample exceeded those requirements.

The study received human subjects research ethics approval by a university institutional review board committee (“Ethikkommission für Forschung in der Psychologie an der Helmut-Schmidt-Universität / Universität der Bundeswehr Hamburg”).

### Survey items

In order to gather data from participants in their daily lives, we used the Participation in Everyday Life (PIEL) Survey App [27]. This app was developed as a tool for ESM by researchers at the University of Sydney with the assistance of Vision Australia. Participants downloaded the PIEL Survey App to their own devices. A control file specifying the study parameters (i.e., the survey questions, the alert sound, the maximum delay, and the sampling times) was loaded into the app. Table 1 displays all items in the order in which they appeared in the ESM questionnaire (S1 Table displays all items in German). At each sampling time, participants responded to questions regarding their most recent aesthetic experience since the previous sampling time. They answered the question “Have you had an aesthetic experience since the last sampling time?” using a binary *no/yes* scale. If they responded *yes*, they were asked to report the content of the aesthetic experience by choosing one of eight categories (visual art, performing art, music, literature, nature, humans, inanimate object, other) and also to report how long ago that aesthetic experience occurred (in minutes). Regardless of whether they reported an aesthetic experience, participants were asked how they had spent their time since the last sampling (at home, outside, alone, with company).

**Table 1:** Experience sampling variables and questions.

Variable	Experience-sampling questions
1 Aesthetic experience^a^	Have you had an aesthetic experience since the last measurement time?
[Items 2 through 11 were presented if the subject reported having an aesthetic experience]
2 Content^b^	What was the content of the aesthetic experience?
3 Time	When did the aesthetic experience occur?
4 Savoring	During the aesthetic experience I savored the present moment.
5 Savoring	During the aesthetic experience I was thinking about things that make me feel happy.
6 Savoring	During the aesthetic experience I was thinking about things that make me feel pleasure.
7 Working memory load (second task)^a^	I was performing some task when the aesthetic experience occurred.
[Items 8 through 11 were presented if the subject reported performing a second task]
8 Challenging	What I was doing was challenging.
9 Interesting	I was interested in what I was doing.
10 Concentrating	I was concentrating on what I was doing
11 Important	What I was doing is important to me.
12 Working memory load (overall level)^c^	How busy have you been since the last sampling time?
13 Context^d^	How have you spent your time since the last sampling?

*Note*. All savoring questions were answered on a seven-point Likert scale (1 = *not at all*, 7 = *very much*). All task characteristic items were answered on a five-point Likert-type scale (1 = *not at all*, 5 = *very much*).

^a^Binary variable (*yes*/*no*).

^b^Response options: *visual art, performing art, music, literature, nature, humans, inanimate object, other*.

^c^This question was answered on a seven-point Likert scale (1 = *not at all*, 7 = *very much*).

^d^Response options: *at home, outside, alone, with company*.

#### Savoring of aesthetic experiences

The criterion variable *savoring* was assessed with three items following the phrase “During the aesthetic experience…” (“I savored the present moment,” “I was thinking about things that make me feel happy,” “I was thinking about things that make me feel pleasure”) on a seven-point Likert scale ranging from 1 (*not at all*) to 7 (*very much*). These items were used in a previous study [28]. Cronbach’s alpha for the savoring scale (calculated across all reports and participants) was *α* = .86.

#### Working memory resources

We assumed that working memory resources were reduced if participants were engaged in a secondary task when the aesthetic experience occurred.

To test this assumption, the item “I was performing some task when the aesthetic experience occurred” was assessed with a binary *no/yes* scale.

If the answer was *yes*, the items “What I was doing was challenging,” “I was interested in what I was doing,” “I was concentrating on what I was doing,” and “What I was doing is important to me” were answered on a five-point Likert scale ranging from 1 (*not at all*) to 5 (*very much*). These items were used in an ESM study on mind wandering [23].

At the end of the questionnaire, regardless of whether they had reported an aesthetic experience, participants were asked about their perceived overall working memory load since the last sampling time (“How busy have you been since the last sampling time?”), which was answered on a seven-point Likert scale ranging from 1 = *not at all* to 7 = *very much*).

### Procedure

Data were collected in June 2020 under social distancing conditions, three months after the first outbreak of the coronavirus pandemic in Germany. Prior to the actual study, we conducted a 4-day pilot test with three volunteers in order to assure feasibility. Before the actual study began, all participants received information about the study procedure and gave us their written informed consent and demographic information (gender, age, study program). They received a 30-min training session via telephone before the start of the study. During the training, a research assistant defined the concept of aesthetic experience in line with previous work [1, 14, 29] as follows (see Instruction in the S1 Appendix for the German version):

An aesthetic experience is the reception or evaluation of an object or sensorial entity with respect to one or more relevant concepts (such as beauty, elegance, rhythm, and so forth).

Participants were then asked to identify an example of an aesthetic experience that happened to them during the last 24 hours. Next, the participants were instructed to download the PIEL Survey App, and the entire procedure and all items were explained in detail, ensuring that every step of the study procedure as well as every item was correctly understood. At the end of the training, participants completed a sample questionnaire for practice.

During the following 14 days, participants were randomly prompted by the mobile app twelve times a day during individually chosen 60-min time blocks to fill out the questionnaire. This frequency was chosen because it is unclear how often aesthetic experiences occur in everyday life—we assumed that sampling individuals’ experience twelve times during their waking hours would increase the odds of observing aesthetic experiences and would also be bearable and not too disruptive to their day. The participants then had up to 20 min to initiate their response and up to 10 min to complete each question. The order of savoring items and task characteristic items was randomized.

After one week, participants received an interim call from the research assistant. They were asked about any questions that might have arisen during the first week. This call was also intended to ensure participants’ compliance.

At the end of the 14 days, the research assistant made a final call to participants. They were asked about the development of their motivation to fill out the questionnaires, how laborious and annoying they judged the study to be, and the reasons for any omitted questionnaires. This call was also intended to ensure the quality of the data and to uncover the participants’ reasons for missing data and omitted questionnaires.

### Statistical analyses

Data were analysed using IBM Statistics SPSS for Mac, version 25 (IBM Corp., Armonk, NY, USA). Due to the hierarchical structure of the ESM data (i.e., questionnaire responses at Level 1 are nested within subjects at Level 2), we employed multilevel modeling (MLM). In contrast to ordinary least squares (OLS) regression, multiple error terms are used in MLM to partition the variance between the levels in the data [30]. Thus, relationships both within and between levels can be analyzed without violating standard assumptions of independence. Multilevel models are fitted to the data using maximum likelihood estimation. This approach is able to cope well with missing and unbalanced data.

In our analyses, the Level 1 variables were group-mean centered, while the Level 2 variables were grand-mean centered. For each analysis, we constructed parallel models, following a general “build-up” strategy for model testing [31]: In the first step, we used a null model (i.e., a model with no predictors) in order to determine whether there is sufficient variability in the sample in the intercepts at Level 2 (across participants). In the second step, we added the Level 1 predictor. Since variance between groups had been excluded by group-mean centering, we reintroduced the mean of the predictors in the third step of our model testing. For this purpose, we entered the mean of the Level 1 predictor as a predictor variable on Level 2. By doing so, we were able to investigate separate within-group and between-group effects of the predictors. For all analyses, we set the conventional.05 alpha level.

## Results

### Overall rate and content of aesthetic experiences

The overall rate of aesthetic experiences reported on the experience sampling questionnaires over the course of two weeks was, on average, 35% (*SD* = 20%, range = 3%–94%). Aesthetic experiences arose most frequently in response to nature (22%), followed by human beings (20%), performing arts (16%), inanimate objects (15%), music (13%), literature (5%), visual arts (2%), and other (6%). During 35% of the aesthetic experiences, participants were also occupied with a second task.

### Hypothesis 1: Does a working memory load lead to fewer aesthetic experiences?

#### Model 1: Association between working memory load and the probability of reporting an aesthetic experience

Do higher levels of working memory load since the previous sampling time reduce the probability of reporting an aesthetic experience? In order to test this assumption, we constructed a two-level model for binary outcome variables. The standard assumption is that the outcome variable has a Bernoulli distribution. The model can then be written as follows:

$$\begin{array}{c} log\left[\frac{p_{ij}}{\left(1-p_{ij}\right)}\right]=\beta_{0j}+\beta_{1j}X_{ij}+r_{ij}, \end{array}$$ (1)

where *i* represents the i^th^ assessment point and *j* represents the j^th^ participant; *p*_*ij*_ is the probability of reporting an aesthetic experience; *β*_0*j*_ denotes the intercept (i.e., the average probability of reporting an aesthetic experience for participant *j*); ***X***_***ij***_ denotes the predictor working memory load; *β*_1*j*_ is the unstandardized coefficient representing the relationship between working memory load and aesthetic experiences for participant *j*; ***r***_*ij*_ is the Level 1 random effect. In this analysis, there were 14,614 observations nested within 115 individuals.

First, we wanted to establish the extent to which the odds of reporting an aesthetic experience varies from one participant to another. In order to do so, we ran a null model and calculated the intraclass correlation coefficient (ICC). The ICC quantifies the degree of homogeneity of the outcome between participants.

$$\begin{array}{c} ICC=\frac{\sigma^{2}}{\left(\sigma^{2}+\left(\frac{\pi^{2}}{3}\right)\right)}=\frac{1.04}{1.04+3.29}=0.24. \end{array}$$ (2)

In Eq 2, *σ*^2^ is the random intercept variance—that is, the Level 2 variance component, and $\frac{\pi^{2}}{3}=3.29$ is the standard logistic distribution—that is, the assumed Level 1 variance component. We used this assumed value because the logistic regression model does not include a Level 1 residual. The result showed that 24% of the chances of having an aesthetic experience were explained by between-person differences.

Second, we included the Level 1 predictor of overall working memory load. In order to interpret the coefficient *β*, we raised it to the exponent to obtain an *OR*. Formally, the *OR* refers to the multiplicative factor by which the predicted probability of reporting an aesthetic experience changes when the predictor working memory load increases by one unit. We found that *β* = −.53, *exp*(*β*) = *OR* = .59, and the 95% confidence interval (CI) = [.54,.64]. Since the 95% CI for the *OR* did not include 1, the effect was statistically significant. Congruent with Hypothesis 1, this indicates that a higher overall working memory load was associated with fewer aesthetic experiences. Adding the Level 2 predictor of mean overall working memory load per subject also yielded a statistically significant outcome (*β* = −.25, *OR* = .78, 95% CI = [.62,.98]), indicating that participants who experienced higher levels of overall working memory load reported fewer aesthetic experiences.

### Hypothesis 2: Does a working memory load reduce the savoring of aesthetic experiences?

#### Model 2a: Association between the presence of a second task and the savoring of aesthetic experiences

To test whether a second task would lead to reductions in savoring ratings, we constructed a random coefficient model. Eq 3 shows how—at Level 1—the savoring rating ***Y***_*ij*_ that person *j* gave at assessment point *i* is being predicted by varying intercepts *β*_0*j*_ and the predictor second task (***X***_***ij***_). The coefficient *β*_1*j*_ is the slope relating the presence of a second task for person *j* to the savoring rating; ***r***_*ij*_ denotes the residual error for assessment point *i* and person *j*.

$$\begin{array}{c} Y_{ij}=\beta_{0j}+\beta_{1j}X_{ij}+r_{ij}, \end{array}$$ (3)

**Fig 1:**
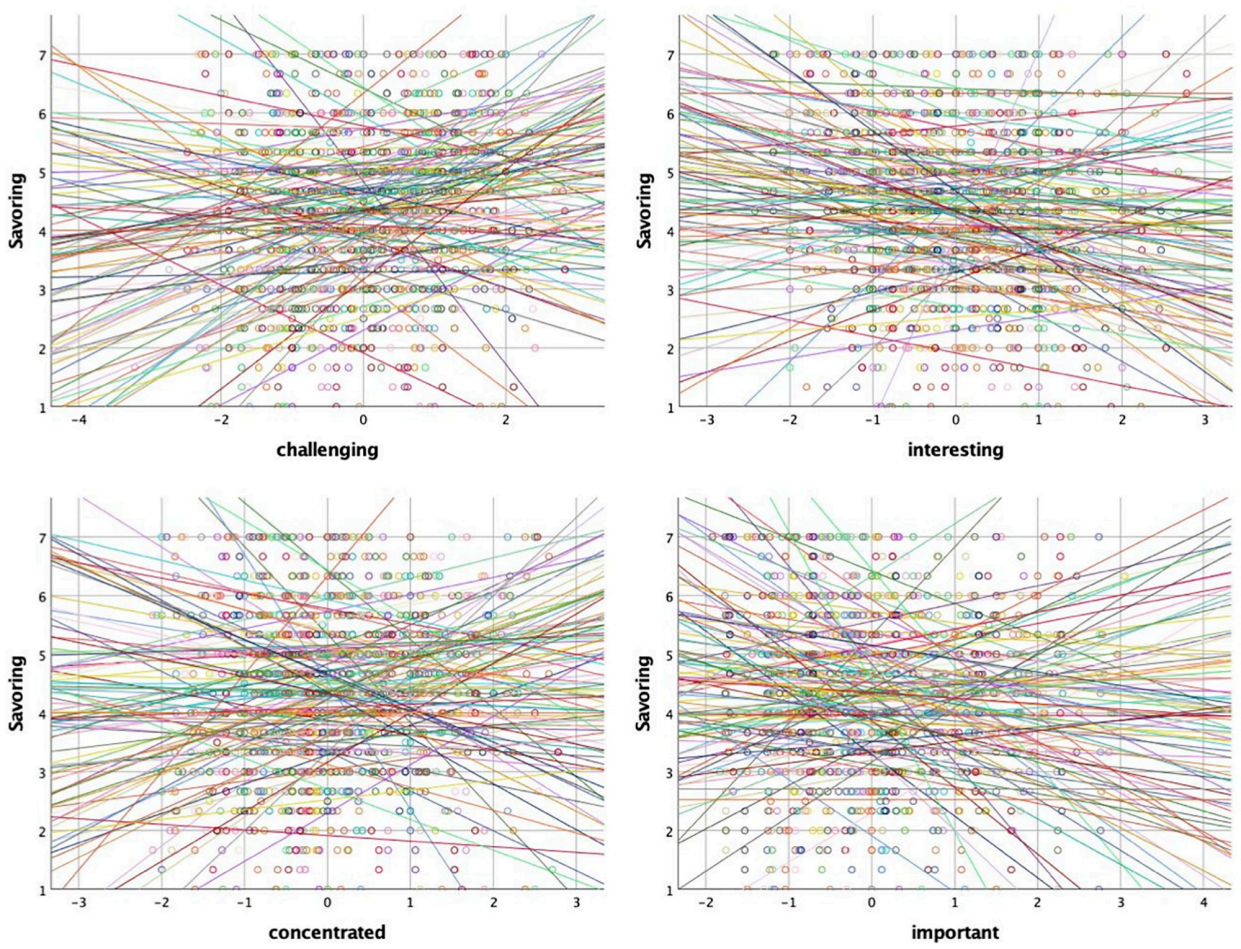
Relationship between savoring of aesthetic experiences and task characteristics for each participant (*N* = 115). The x-axes denote the standardized values for the task characteristics items *challenging, interesting, concentrated, important*. Each color-coded line represents the slope of an individual participant.

Eqs 4 and 5 refer to Level 2. For this model as well as Model 2b and Model 3, the intercept was specified to be randomly varying at the participant level, thus accounting for the fact that observations tend to be more similar if they are taken from the same person. Eq 4 shows how person *j*’s intercept *β*_0*j*_ (i.e., the average savoring value) is composed of the overall intercept *γ*_00_ and a Level 2 error term *μ*_0*j*_. For theoretical reasons and based on graphical inspections of the regression slopes (see Fig 1 for the task characteristics), all predictors were modeled with coefficients that randomly varied at the participant level, allowing the relationship between each predictor and the outcome measure to differ between individuals. Eq 5 shows how the second task slope for person *j* is composed of the overall slope *γ*_10_ and a Level 2 error term *μ*_1*j*_.

$$\begin{array}{c} \beta_{0j}=\gamma_{00}+\mu_{0j} \end{array}$$ (4)

$$\begin{array}{c} \beta_{1j}=\gamma_{10}+\mu_{1j} \end{array}$$ (5)

Again, we first computed a null model and calculated the ICC in order to assess the exted to which the savoring values vary between participants. The null model comprised only the mean of the savoring scale as the dependent variable, with the savoring ratings of each participant in the intercept. The ICC was calculated as.25, thus estimating that 25% of the savoring variance is explained by the differences between participants, which suggests substantial clustering in the data. This finding supported the use of random coefficient modeling rather than OLS regression to analyze the data.

In the second step, we entered the values of the dummy-coded variable *second task* (Level 1 predictor). In the third step, the proportion of second tasks performed by each individual (Level 2 predictor) was added. Table 2 presents the results. In order to assess whether the addition of the predictors improved the fit of our model, we used a goodness of fit test known as a likelihood ratio (LR) chi-square difference test. LR tests are used to compare nested models, wherein a null model or reduced model is compared to another model with additional parameters of interest. We found that a second task was associated with lower savoring values, and inclusion of the Level 1 predictor significantly improved the model fit, with a reduction in the log-likelihood, *χ*^2^ = 255.27, *df* = 3, *p* <.001. Inclusion of the Level 2 predictor resulted in a poorer model fit, as indicated by a significant increase in the log-likelihood, *χ*^2^ = −20.02, *df* = 4, *p* <.001. Thus, as hypothesized, the presence of a second task was significantly associated with a decrease in the savoring of aesthetic experiences.

**Table 2:** General linear mixed models for savoring value.

Model 2 (a) Savoring and second task					
	Intercept	Second task			
Estimate	4.76	-.59			
CI lower	4.61	-.70			
CI upper	4.91	-.48			
*SE*	.75	.06			
*t*-value	63.87	-10.62			
Model 2 (b) Savoring and overall load					
	Intercept	Overall load			
Estimate	4.60	-.12			
CI lower	4.45	-.16			
CI upper	4.76	-.08			
*SE*	.08	.02			
*t*-value	59.96	-.5.35			
Model 3 Savoring and task characteristics					
	Intercept	demanding	interesting	concentrating	important
Estimate	4.34	.15	-.22	.11	-.08
CI lower	4.17	.07	-.30	.01	-.18
CI upper	4.50	.24	-.15	.21	-.00
*SE*	.08	.04	.03	.05	.04
*t*-value	52.51	3.65	-5.70	2.20	-1.97

*Note*. Dependent variable: *savoring*; fixed effects: second task, proportion second task (Model 2a), overall load (Model 2b), task characteristics (Model 3); random effects: intercept. Age, gender, group means of overall cognitive load, and group means of task characteristics were excluded from the analyses after they were found out to have no effect.

#### Model 2b: Association between perceived overall working memory load and the savoring of aesthetic experiences

To test whether the perceived overall working memory load impaired the savoring of aesthetic experiences, we computed another random coefficient model. In the second step, we added perceived overall working memory load (Level 1 predictor). In the third step, we added individual means of the overall working memory load (Level 2 predictor). Table 2 presents the results. When the Level 1 predictor overall working memory load was added, the model fit improved significantly, as can be seen in the reduced log-likelihood, *χ*^2^ = 1505.15, *df* = 3, *p* <.001. In contrast, adding the individual means of the overall working memory load resulted in a poorer model fit, as can be seen in the increased log-likelihood, *χ*^2^ = −237.63, *df* = 4, *p* <.001. Thus, higher levels of perceived overall working memory load led to a significant reduction in the levels of savoring.

### Hypothesis 3: Do certain task characteristics impair aesthetic experiences?

#### Model 3: Association between task characteristics and the savoring of aesthetic experiences

To test whether certain task characteristics (challenging, interesting, requiring concentration, important) lead to a reduction in the savoring of aesthetic experiences, we computed another random coefficient model. In the second step, task characteristics were included. In the third step, individual means of task characteristics were added. The results are displayed in Table 2. Adding the Level 1 predictors significantly improved the model fit (*χ*^2^ = 11122, *df* = 18, *p* <.001). Adding the Level 2 predictors did not further improve the model fit (*χ*^2^ = 4.40, *df* = 5, *p* = .493). Thus, in line with Hypothesis 3, second tasks that were perceived as interesting (*β* = −.22, *SE* = .04, *t* = −5.70, *p* <.001) and important (*β* = −.09, *SE* = .04, *t* = −1.97, *p* = .049) impaired savoring. In contrast, if a second task was perceived as demanding (*β* = .15, *SE* = .04, *t* = 3.65, *p* = .001) and required a lot of concentration (*β* = .11, *SE* = .05, *t* = 2.20, *p* = .032), the savoring of aesthetic experiences was enhanced.

### Contextual correlates of aesthetic experiences

To test whether the probability of having an aesthetic experience was affected by the context variables (at home, outside, alone, with company), we employed another multilevel binary logistic regression. We included the Level 1 context predictors and computed the *OR*. We found that for the contexts *alone* (*β* = −.61, *OR* = .54, 95% CI [.39,.76]) and *at home* (*β* = −.80, *OR* = .45, 95% CI [.28,.71]), the effect was statistically significant since the 95% CI did not include 1. In Table 3, we summarize the multilevel binary logistic regression model to estimate the effects of the context on the likelihood of having an aesthetic experience. The predictors *outside* and *with company* were not significant. Including the Level 2 predictors of the percentage of time spent at home, outside, alone, or with company per subject did not result in significant outcomes (all CIs included 1). Thus, subjects were less likely to have an aesthetic experience when they were at home or alone.

**Table 3:** Log odds estimates (*N* = 15, 047).

Parameter	*β*	*SE*	*p*	*Exp*(*β*)	95% CI *Exp*(*β*)	
					Lower	Upper
(Intercept)	.29	.34	.401	1.33	.68	2.59
At home	-.80	.23	.001	.45	.28	.71
Outside	.05	.21	.809	1.05	.69	1.60
Alone	-.61	.17	<.001	.54	.39	.76
With company	-.26	.19	.167	.77	.54	1.11

*Note*. Dependent variable: *aesthetic experience*; model: (intercept), at home, outside, alone, with company.

We computed another random coefficient model in order to test whether the context variables affected the intensity of savoring of aesthetic experiences. After entering the Level 1 predictors, *at home* (*β* = −.29, *SE* = .06, *t* = −4.55, *p* <.001), *alone* (*β* = −.19, *SE* = .06, *t* = −3.25, *p* = .001), and *with company* (*β* = .15, *SE* = .06, *t* = 2.48, *p* = .013) proved to be significant predictors of savoring. Adding the Level 2 predictors did not improve the model fit (*χ*^2^ = 6.32, *df* = 4, *p* = .176). Thus, subjects did savor aesthetic experiences less when they were at home or alone, and they savored aesthetic experiences more when they were with company.

## Discussion

We studied aesthetic experiences in everyday life through ESM for a period of two weeks. The key objective of the study was to conduct an ecologically valid investigation of the role that working memory resources play in everyday aesthetic experiences across various domains and qualities of aesthetic experiences.

First, as hypothesized, we found that the frequency of aesthetic experiences was negatively predicted by participants’ perceived overall working memory load. Also consistent with our predictions, the presence of a second task and higher levels of perceived overall working memory load were associated with reduced savoring of aesthetic experiences. These results suggest that in everyday life, limited working memory resources impair the frequency and intensity of aesthetic experiences. This finding is in line with theory that points to the necessity of concentrating working memory resources on the aesthetic stimulus in order to maximize the aesthetic experience [8, 13–15], as the aesthetic experience stands in conflict with other goal-oriented behavior [9]. Experimental work [17, 18] also points in a similar direction.

Second, we investigated whether task demands that make it easier to sustain attentional focus on the task at hand would further impair savoring of aesthetic experiences. In that vein, we found that interesting or important second tasks impaired the savoring of aesthetic experiences. In prior research, interesting tasks have been shown to draw on greater working memory resources than less interesting tasks [32].

In contrast, second tasks that were perceived as demanding or required concentration were associated with higher savoring values. Since aesthetic distraction has not yet been studied in laboratory research, this finding points to the possibility that certain predictions based on previous work will not hold true for the quality of aesthetic distraction. In order to elicit aesthetic experience, a stimulus must exceed the beholder’s aesthetic threshold [33, 34]. It is possible that only stimuli with comparatively high aesthetic appeal can distract people from a challenging and concentration-demanding task because other stimuli don’t reach the threshold. Another explanation for this finding could lie in different categories of motivation for interesting/important versus challenging/attention-demanding tasks. Speculatively, interesting and important tasks (note the wording of the item: “[…] important *to me*”) could have been associated with an intrinsic motivation, whereas tasks that were rated high in terms of being challenging and requiring concentration might instead have been extrinsically motivated. Intrinsically motivated tasks that match the individual’s personal skill level can result in a flow state that is associated with effortless attention to the task and lower proneness to distractions [35]. It is probable that during intrinsically motivated tasks, aesthetic distractions are also less intense. In line with this, evidence suggests that individuals’ minds tended to wander more when they perceived a task as stressful [36]. Hence, engaging in aesthetic experiences could have been a means to cope with stressful tasks. To shed light on these issues, future studies that specifically investigate the quality of aesthetic distraction would be valuable.

Our study’s findings may also have a number of implications for advancing our understanding of the cognitive basis of aesthetic experiences. The findings provide novel and highly ecologically valid empirical data consistent with prior research that was mostly conducted in the art domains. The present study therefore completes the picture by allowing not only generalization across a variety of aesthetic domains, but also across different qualities of aesthetic experiences. Our finding that aesthetic experiences are savored less and occur less frequently when working memory resources are depleted should be considered with regard to possible consequences for emotion regulation, health, and well-being, as temporary experiences of positive emotions are known to promote a variety of personal resources, such as resilience [37]. Specifically, savoring of aesthetic experiences has been shown to contribute to well-being and life satisfaction, as well as less negative affect and depression [38–40]. Aesthetic feelings can be instrumental for mood regulation and may even promote long-term emotional capacities [41]. Therefore, interventions to help people leave their daily concerns behind could enhance the frequency and intensity of aesthetic experiences, thereby contributing to improved emotional capacities and greater life satisfaction. Also, consistent with the “stopping for knowledge” hypothesis [9], our findings suggest that it may be important to not reduce aesthetic experiences to a merely decorative aspect of life experience that can happen any time, but rather to consider aesthetic experiences as a key part of our knowledge acquisition process, thereby requiring working memory resources.

Finally, it is important to acknowledge the scope of this investigation. Measurement of the studied phenomena might have been limited by the trade-off between capturing the phenomena comprehensively and ensuring that the sampling was brief enough to allow high temporal resolution and reduce the amount of missing data. We tried to mitigate this problem by using a previously employed savoring scale instead of a one-item measurement and by employing two ways to infer current working memory resources (i.e., perceived overall working memory load and a second task). However, these measurements rely on self-reports and may therefore be subject to demand characteristics, even though the final phone calls revealed no correct hunches about the hypothesis. We acknowledge that measuring working memory resources in an ESM study is a challenging endeavor. Traditional means of measuring “working memory capacity” (e.g., complex span tasks, updating tasks; [42]) have the disadvantage of interfering with ecological validity since they directly interfere with the flow of everyday life and are unrelated to the individual’s current experience. Our measurement of working memory resources is based on the idea that, in a limited-capacity system, task-irrelevant distractors (i.e., aesthetic stimuli) compete with task-relevant demands (e.g., learning, cleaning, talking to friends) for working memory resources [43]. Also, we acknowledge that answering a questionnaire—even a short one—also interferes with the flow of everyday life and affords working memory resources. Over a two-week period, participants were asked to repeatedly remember and evaluate events from the last hour. Therefore, task engagement may have been subject to change. Even though, in the final calls, participants reported low task-specific load, future studies may assess subjective changes in task-engagement over the course of the study in a more reliable and systematic way. We have to keep in mind that it is very hard—especially in ESM—to assess task-engagement or the potential reactivity of the tasks given to participants [44] since this would usually involve administering yet another task. The challenge here is to find the right balance between capturing task engagement on the one hand and keeping the daily questionnaires as short as possible, on the other. Overly burdensome questionnaires or a high frequency of assessments could result in a state of mental fatigue which is characterized by an inability to allocate sufficient working memory resources to the current task [45]. This state may affect all stages of information processing that receive modulatory top-down input [46], it might impair task engagement and—ultimately—the validity of the responses. Ideally, future investigations may apply a multi-method approach of field and laboratory studies where methodological strengths and limitations complement each other in order to see if results converge. Finally, since obtaining reliable measurements is a precondition for obtaining valid measurements, we want to give the issue of reliability some consideration. Common reliability measures (e.g., Cronbach’s alpha) were developed in the context of cross-sectional studies [47]. Since within-subject fluctuations are treated as measurement error in cross-sectional studies, applying standard measurements of reliability to ESM can be problematic. Promising approaches to assessing between-subject and within-subject reliabilities in ESM measures have been developed [48]. Though those methods are not routinely used in ESM, they may provide a more accurate estimate of reliability and should be applied in future studies.

We note that the frequency of aesthetic experiences during the current coronavirus pandemic must surely have shifted towards nonsocial experiences. At the time of our data sampling, socially enclosed opportunities for aesthetic experiences (e.g., cinema, concerts) were close to nonexistent. However, there was no need to refrain from aesthetic experiences, because people do have the capability to voluntarily switch into the aesthetic mode [5]. As a matter of fact, the current pandemic forces many of us to cut back on most activities and to remain at home in a perceptually restricted environment. This further underscores the need to smooth the way for positive (perceptual) experiences in order to promote resilience.

## Conclusion

In summary, we conducted an intensive ESM study and, under the maximally noisy conditions of real life, found confirmation for our hypotheses that a depletion of working memory resources impairs the frequency and savoring of aesthetic experiences. To our knowledge, this is the first study to establish the prevalence of this trade-off in everyday life. We believe that further understanding of the contribution of working memory to the specific qualities of aesthetic experiences may prove important for gaining a holistic understanding of the cognitive preconditions and processes that lead from an input stimulus to the specific outcome of an aesthetic experience.

## Supporting information

S1 Appendix German instruction. (PDF)

S1 Table Experience-sampling questions in German. All savoring questions were answered on a seven-point Likert scale (1 = *überhaupt nicht*, 7 = *voll und ganz*). All task characteristic items were answered on a five-point Likert-type scale (1 = *lehne stark ab*, 5 = *stimme stark zu*). ^a^Binary variable (*ja*/*nein*).^b^Response options: *Bildende Kunst, Musik, Darstellende Kunst, Literatur, Natur, Menschen, unbelebtes Objekt, Sonstiges*.^c^This question was answered on a seven-point Likert scale (1 = *überhaupt nicht*, 7 = *voll und ganz*). ^d^Response options: *zuhause, draußen, allein, in Gesellschaft*.
(PDF)
