# Supplementary material for: Beauty and the busy mind: Occupied working memory resources impair aesthetic experiences in everyday life
Source: PLoS One. 2021 Mar 12;16(3):e0248529. doi: 10.1371/journal.pone.0248529 (PMC7954329; doi:10.1371/journal.pone.0248529)
Supplement: S1 Appendix — (PDF) [file pone.0248529.s001.pdf]

German instruction

During the training, a research assistant defined the concept of aesthetic experience as follows:

> Eine ästhetische Erfahrung ist die Rezeption oder Bewertung eines Objektes oder einer sensorisch wahrnehmbaren Entität im Hinblick auf ein oder mehrere relevante Konzepte (z.B., Schönheit, Eleganz, Rhythmus, usw.).
