# Supplementary material for: Beauty and the busy mind: Occupied working memory resources impair aesthetic experiences in everyday life
Source: PLoS One. 2021 Mar 12;16(3):e0248529. doi: 10.1371/journal.pone.0248529 (PMC7954329; doi:10.1371/journal.pone.0248529)
Supplement: S1 Table — All savoring questions were answered on a seven-point Likert scale (1 = überhaupt nicht, 7 = voll und ganz). All task characteristic items were answered on a five-point Likert-type scale (1 = lehne stark ab, 5 = stimme stark zu). aBinary variable (ja/nein).bResponse options: Bildende Kunst, Musik, Darstellende Kunst, Literatur, Natur, Menschen, unbelebtes Objekt, Sonstiges.cThis question was answered on a seven-point Likert scale (1 = überhaupt nicht, 7 = voll und ganz). dResponse options: zuhause, draußen, allein, in Gesellschaft. (PDF) [file pone.0248529.s002.pdf]

Table S1

Experience-sampling questions in German.

**Variable**	**Experience-sampling questions**
1 Aesthetic experience[a]	Hatten Sie eine ästhetische Erfahrung seit dem letzten Messzeitpunkt?
[Items 2 through 11 were presented if the subject reported having an aesthetic experience]	
2 Content[b]	Worauf bezog sich die ästhetische Erfahrung?
3 Time	Wie lang ist diese ästhetische Erfahrung her (in Minuten)?
4 Savoring	Während der ästhetischen Erfahrung habe ich den Augenblick ausgekostet.
5 Savoring	Während der ästhetischen Erfahrung habe ich an etwas gedacht, was mich glücklich macht.
6 Savoring	Während der ästhetischen Erfahrung habe ich an etwas gedacht, was mir Freude bereitet.
7 Cognitive load (second task)[a]	Ich war mit einer anderen Aufgabe beschäftigt, als ich die ästhetische Erfahrung hatte.
[Items 8 through 11 were presented if the subject reported performing a second task]	
8 Challenging	Die Aufgabe war herausfordernd.
9 Interesting	Die Aufgabe hat mich interessiert.
10 Concentrating	Ich habe mich auf die Aufgabe konzentriert.
11 Important	Die Aufgabe war wichtig für mich.
12 Cognitive load (overall level)[c]	Wie ausgelastet/beschäftigt waren Sie seit dem letzten Messzeitpunkt?
13 Context[d]	Wie haben Sie die Zeit seit dem letzten Messzeitpunkt verbracht?

All savoring questions were answered on a seven-point Likert scale (1 = *überhaupt nicht*, 7 = *voll und ganz*). All task characteristic items were answered on a five-point Likert-type scale (1 = *lehne stark ab*, 5 = *stimme stark zu*). [a]Binary variable (*ja/nein*).[b]Response options: *Bildende Kunst, Musik, Darstellende Kunst, Literatur, Natur, Menschen, unbelebtes Objekt, Sonstiges.* [c]This question was answered on a seven-point Likert scale (1 = *überhaupt nicht*, 7 = *voll und ganz*). [d]Response options: *zuhause, draußen, allein, in Gesellschaft.*.
